# Differentiation of Human Mesenchymal Stem Cells from Wharton’s Jelly Towards Neural Stem Cells Using a Feasible and Repeatable Protocol

**DOI:** 10.3390/cells9030739

**Published:** 2020-03-17

**Authors:** Ewa Kruminis-Kaszkiel, Adam Osowski, Ewa Bejer-Oleńska, Mariusz Dziekoński, Joanna Wojtkiewicz

**Affiliations:** 1 Department of Pathophysiology, School of Medicine, Collegium Medicum, University of Warmia and Mazury, 10-082 Olsztyn, Poland; adam.osowski@uwm.edu.pl (A.O.); ewa.bejer-olenska@wp.pl (E.B.-O.); joanna.wojtkiewicz@uwm.edu.pl (J.W.); 2 Department of Animal Anatomy and Physiology, Faculty of Biology and Biotechnology, University of Warmia and Mazury, 10-719 Olsztyn, Poland; mariuszdziekonski@gmail.com

**Keywords:** neural stem cells, neural progenitor cells, monolayer culture, mesenchymal stem cells, Wharton’s jelly, flow cytometry

## Abstract

The transplantation of neural stem cells (NSCs) capable of regenerating to the cells of the central nervous system (CNS) is a promising strategy in the treatment of CNS diseases and injury. As previous studies have highlighted mesenchymal stem cells (MSCs) as a source of NSCs, this study aimed to develop a feasible, efficient, and reproducible method for the neural induction of MSCs isolated from Wharton’s jelly (hWJ-MSCs). We induced neural differentiation in a monolayer culture using epidermal growth factor, basic fibroblast growth factor, N2, and B27 supplements. This resulted in a homogenous population of proliferating cells that expressed certain neural markers at both the protein and mRNA levels. Flow cytometry and immunocytochemistry confirmed the expression of neural markers: nestin, sex-determining region Y (SRY) box 1 and 2 (SOX1 and SOX2), microtubule-associated protein 2 (MAP2), and glial fibrillary acidic protein (GFAP). The qRT-PCR analysis revealed significantly enhanced expression of *nestin* and *MAP2* in differentiated cells. This study confirms that it is possible to generate NSCs-like cells from hWJ-MSCs in a 2D culture using a practical method. However, the therapeutic effectiveness of such differentiated cells should be extended to confirm the terminal differentiation ability and electrophysiological properties of neurons derived from them.

## 1. Introduction

Neurodegenerative diseases cause considerable damage to the central nervous system (CNS) such as loss of sensation, motor function, or memory. Although the causes of neurodegenerative diseases are multifactorial and the pathological mechanisms underlying the diseases are complex, the basic feature of all forms of neurodegeneration is a progressive loss in function and number of neurons and glia [1,2]. Moreover, neurodegenerative pathologies are often very difficult to treat and constitute a direct cause of death in patients. The pathogenesis of neurodegenerative diseases is still not fully elucidated and traditional therapy can only slow disease progression. The current available pharmacological and neurosurgical therapies provide only transient benefits and do not arrest the progression of the neuropathological changes [1,3]. Therefore, to recover and restore lost cells and their function, a regenerative approach is needed that can prolong the lifespan and improve cognitive, motor, and sensory deficiencies in patients suffering from neurodegenerative diseases [4,5,6]. Stem cell therapy enables the regeneration of neural tissue by restoring lost cells and providing neurotrophic support for existing cells [3]. Thus, a therapy that employs transplantation of stem cells and progenitor cells is a promising strategy in the treatment of various diseases of the CNS [7,8]. However, the type and origin of the exogenous stem cells are crucial in the context of its therapeutic efficacy. It appears that cells that are directed to neural lineage have a greater potential to differentiate towards functional neurons and glia [7].

Recent studies have revealed that the transplantation of neural stem cells (NSCs) capable of regenerating to the nervous system is a promising and precise approach in the therapy of CNS diseases and injuries [8,9]. Many clinical trials have been registered in which NSCs are tested to treat neurodegenerative diseases such as amyotrophic lateral sclerosis (ALS), multiple sclerosis (MS) Parkinson’s disease (PD), Alzheimer’s disease (AD), spinal cord injury (SCI), and even tumors such as gliomas [10]. Certainly, each CNS disease requires a tailored approach to effectively restore the neuronal networks. NSCs can be used in cell-replacement strategies, including replacement of dysfunctional astroglia or degenerated motor neurons in ALS [11] or oligodendrocyte replacement in MS [12]. Cell-based neurotrophic support, another regenerative approach, has been widely applied to target a broad spectrum of CNS diseases [3]. Neurotrophic growth factors (NTF) produced by NSCs provide trophic support to dysfunctional neuronal populations and synapses and have been exploited in neurodegenerative diseases [3,12]. Moreover, NSCs can be genetically modified and used as carriers to deliver therapeutics such as neprilysin, a proteolytic enzyme involved in the degradation of amyloid-beta (Aβ) plaques to treat AD [13,14] or cytosine deaminase, which converts the prodrug 5-fluorocytosine (5-FC) to 5-fluorouracil (5-FU) to treat glioma [15]. NSCs are self-renewable multipotent stem cells, able to differentiate towards both neurons and glia through a multistep process [9]. During the process of neurogenesis, NSCs undergo several stages, including neural progenitor cells (NPCs), neuroblasts and mature neurons, oligodendrocytes, and astrocytes [16]. NSCs undergo self-renewing asymmetric cell division and give rise to additional NSCs and a lineage-committed cell in contrast to their non-stem cell progeny—NPCs that have very limited self-renewal and proliferative capacity [17]. NPCs can give rise to either neurons or glia and their fate is niche-dependent [16,18]. Hence, the main difference between NSCs and NPCs is that NPCs are lineage-committed and are capable of generating only one category of neural component, either glial cells or neurons [17]. Neurons are specialized cells containing: a cell body, dendrites, and an axon with its terminals. They receive and transmit chemical or electrical signals [19]. Glial cells (astrocytes, oligodendrocytes) play a fundamental role in the maintenance of the blood-brain barrier, neuronal survival and support, synapse formation and their strength, and are involved in myelination [20,21]. Indeed, supportive glial cells can respond to neural injury and might improve functional recovery [3]. NSCs, NPCs, and mature cells that originate from them (neurons, astrocytes, and oligodendrocytes) can be characterized based on their morphology and expression of specific markers. Since it is difficult to use a single marker to define a cell phenotype, multiple markers can be used [22,23]. Examples of markers that are commonly used to define the phenotype of NSCs and their progeny have been listed in Table 1.

NSCs can be obtained through direct isolation from the brain tissue of a fetus [35] and adult humans [36] or they can be differentiated from pluripotent stem cells including embryonic stem cells (ESCs) [37] and induced pluripotent stem cells (iPSCs) [38]. Embryonic or fetal origin of cells is challenging from a technical point of view and is always related to ethical issues [8,9]. Although using iPSCs as an alternative seems interesting, the reprogramming procedure is time-consuming and the purity of the obtained pluripotent stem cells is disputable [8,39]. Thus, another source of NSCs was recently highlighted—trans-differentiation from multipotent mesenchymal stem cells (MSCs) [40].

In principle, MSCs are multipotent and can differentiate in vitro towards osteoblasts, chondroblasts, and adipocytes. They are defined as cells with fibroblast-like morphology, able to adhere in culture to a plastic surface and express characteristic markers including CD105, CD73, CD90, and a lack of antigens specific for endothelial and hematopoietic lineage (CD45, CD34, CD31, CD14, CD11b, and HLA-DR) [41,42]. The plastic adherence property, the tripotent differentiation capacity, and the expression of specific markers comprise the minimal criteria that are required for defining MSCs according to the International Society for Cellular Therapy [41]. Human MSCs can be isolated from various adult tissues among which bone marrow [43] and adipose tissue [44] are important from a practical point of view and have been widely investigated. MSCs can also be isolated from fetal tissues, including placenta [45], amniotic fluid [46], umbilical cord [47], and umbilical cord blood [48]. MSCs derived from different sources exhibit distinct viability, purity, and differentiation potential [49]. Even though MSCs harvested from various tissue sources meet all three criteria set for MSCs (see above), an increasing number of comprehensive flow cytometry and immunocytochemistry analysis reveal that MSCs exhibit profound differences in their transcriptome profile [50] and protein expression when isolated from different sources [51,52]. Moreover, the origin of MSCs has an influence on the maintenance of expression of specific markers during the cell culture period [52]. The proliferation rate of MSCs of fetal origin is higher compared to cells isolated from adult tissues [51]. In fact, MSCs are a heterogeneous cell population and the profile of cell subsets comprising MSCs influence the differentiation potential of those cells [50].

A significant feature of MSCs is their plasticity; the ability to transdifferentiate towards cells beyond mesodermal origin such as endodermal and ectodermal lineage e.g., cells of the CNS [53,54]. In the context of CNS diseases therapy, the high neurogenic potential of MSCs should be highlighted, as they can differentiate towards NSCs and NPCs both in vitro [55] and in vivo [56]. As mentioned earlier, the source of MSCs influences their differentiation potential and it has been found that MSCs from Wharton’s jelly of the umbilical cord (WJ-MSCs) have higher neurogenic differentiation capacity than MSCs of adult origin [57]. WJ-MSCs express proteins characteristic of NSCs and NPCs including nestin and SOX2 might contribute to their higher neurogenic potential [58,59] and the unique secretome of WJ-MSCs favors neurogenesis and neuroprotection [57,60]. Moreover, indirect neural induction of WJ-MSCs leads to the formation of neurospheres that are bigger compared to neurospheres derived from MSCs isolated from bone marrow and adipose tissue [57].

A subpopulation of undifferentiated WJ-MSCs expresses nestin—a protein marker characteristic of NSCs and NPCs [58,59] and pluripotency markers including SOX2, Nanog, and octamer-binding transcription factor 4 (Oct4) [28], which make them able to differentiate towards neural lineage. The appropriate neural induction leads to altered morphology of the cells and expression of characteristic markers confirming a phenotype shift. Although several different protocols for differentiation of WJ-MSCs towards neural lineage have been developed, there is still a need for the development of a protocol that is simple, reproducible, and is not laborious and time-consuming. Such a protocol could be beneficial in therapeutic approaches involving NSCs in neurodegenerative diseases. A common issue in the therapeutic application of NSCs is an adequate dosage of cells to cause a regenerative effect. The quantity and viability of NSCs are affected by their origin and method of isolation [8]. The obtained cells should comprise a homogenous population that is safe and not immunogenic. Therefore, especially in the context of therapy, it is important to establish a feasible, reproducible, and efficient method of NSCs isolation and culture.

There are two main approaches in the neural differentiation of MSCs, direct and indirect. The indirect method consists of two steps: an initial suspension culture to generate neurospheres and a subsequent differentiation culture to generate cells of a certain phenotype including neurons, oligodendrocytes or astrocytes [61,62]. The procedure is time-consuming and sometimes requires digestion of neurospheres to obtain NSCs. Moreover, neurospheres growing in suspension are heterogeneous structures containing various cell types and the actual percentage of NSCs can be relatively low [63]. Therefore, the direct method that employs a monolayer culture of MSCs appears to be valuable. Despite various direct protocols described in the literature, generating a pure NSCs population is still challenging. Published approaches of neural induction of MSCs in monolayer culture rely on using several different factors such as epidermal growth factor (EGF), N2, and B27 supplements [64]; basic fibroblast growth factor (bFGF), dimethyl sulfoxide (DMSO), and forskolin [65,66]; EGF, bFGF, nerve growth factor (NGF), retinoic and ascorbic acid [67]; forskolin and 3-isobutyl-1-methylxanthine (IBMX) [68]; cerebrospinal fluid [69]; and resveratrol [70]. All of these approaches share a common factor, the addition of serum in culture to facilitate cell adhesion. Although all of these approaches have led to neural induction, the efficiency of these protocols is variable.

Taking advantage of the direct method, the study aimed to develop a simple and efficient protocol for generating NSCs from MSCs isolated from human Wharton’s jelly and to characterize the phenotype of the cells obtained. Based on these approaches and already known utility of EGF and bFGF for maintenance of fetal NSCs in adherent culture [71] we used EGF, bFGF, N2, and B27 supplements, as well as low serum in our protocol. Moreover, in addition to other commonly used methods to characterize differentiated cells, we applied flow cytometry enabling comprehensive qualitative and quantitative analysis of neural markers in the differentiated cell population. This method is rarely used for immunophenotyping of NSCs generated from MSCs and data are usually presented as separate histograms or graphs, probably due to insufficient cell numbers that is required for the procedure [61,72]. Here, we present broad and comprehensive flow cytometry analysis of neural markers in differentiated cells.

## 2. Materials and Methods

### 2.1. Isolation and Culture of MSCs from Human Wharton’s Jelly of the Umbilical Cord (hWJ-MSCs) Using Tissue Explants Method

Ten different human umbilical cords were obtained after caesarean section from healthy donors with the mothers’ written agreement and approved statement of the Ethics Committee of University of Warmia and Mazury in Olsztyn (Poland) (statement date: 27th September 2016). Each umbilical cord unit was stored at 4 °C in sterile containers in phosphate-buffered saline (PBS) containing 1% Penicillin/Streptomycin (Sigma-Aldrich, St. Louis, MO, USA) up to 12 h before tissue processing. Umbilical cord fragments were manipulated under sterile conditions using a class-II biosafety cabinet. Firstly, they were rinsed thoroughly using sterile PBS with antibiotics (Penicillin/Streptomycin, Sigma- Aldrich, St. Louis, MO, USA) to remove blood. After subsequent removal of the vein and arteries, Wharton’s jelly (WJ) was cut into tiny pieces (≤0.5 cm) using sterile scalpel and forceps. Groups of approximately 15 fragments were transferred to each 10 cm Petri dish and MSCs medium (DMEM/F12 with Glutamax (Gibco, Carlsbad, CA, USA)) supplemented with 10% of fetal bovine serum (FBS) (Sigma-Aldrich, St. Louis, MO, USA) and 0.2% Penicillin/Streptomycin (Sigma-Aldrich, St. Louis, MO, USA) was added to cover the fragments. The fragments were then maintained for 7–10 days in the incubator with proper conditions at 37 °C with 5% CO_2_, 21% O_2_, and 95% humidity until MSCs migrating out from WJ fragments formed well-defined colonies. The fragments were then removed from the culture dish and cells were passaged. The cells were dissociated using a 0.25% Trypsin/EDTA solution (Sigma-Aldrich, St. Louis, MO, USA). Trypsin was inactivated using 2× the volume of MSCs medium, centrifuged (200×g, for 10 min at RT), counted, and seeded at a density of 2 × 10^3^ cells/cm^2^ in MSCs medium and cultured in the incubator (as above) until the cells reached 80% confluence. At this stage, the cells were harvested and cryopreserved. For freezing the cells, they were treated in a similar way as for passaging but at the end, the cells were resuspended in a cryopreservation solution (FBS supplemented with 10% DMSO (Sigma-Aldrich, St. Louis, MO, USA)) at a density of 2 × 10^6^ cells/cryovial. The cells were then transferred to a cell-freezing container filled with isopropanol to achieve a slow rate of cooling. For long-term storage, the cryopreserved cells were transferred to a liquid nitrogen cryotank. A total of 5 × 10^5^ cells were cryopreserved in 1mL of TRI Reagent (Sigma-Aldrich, St. Louis, MO, USA) for further RNA isolation.

### 2.2. Immunophenotypic Characterization of hWJ-MSCs

The cryopreserved MSCs isolated from human Wharton’s jelly of the umbilical cord (hWJ-MSCs) generated as described above were thawed in warm DMEM/F12 with Glutamax and centrifuged (200× *g*, for 10 min at RT). The cells were then resuspended in PBS, counted, and stained in polystyrene tubes using fluorescently-labeled antibodies from Human MSC Analysis Kit (Cat. No 562245, BD Biosciences, San Diego, CA, USA).

For compensation, the cells were stained with each antibody conjugated to a different fluorochrome. The phenotype was characterized using a flow cytometer (FACS Aria III, BD Biosciences, San Diego, CA, USA) and Flow Jo LLC V10 software (BD Biosciences, San Diego, CA, USA). The positive and negative markers are listed in Table 2.

### 2.3. Neural Induction of hWJ-MSCs

The cryopreserved hWJ-MSCs were thawed and seeded on poly-L-lysine-coated T75 flasks at a density of 2 × 10^3^ cells/cm^2^ in MSCs medium (Day 0). The cells were cultured in this medium at 37 °C with 5% CO_2_, 21% O_2_, and 95% humidity for one day. After approximately 24 h when the cells were well attached to the surface, the medium was changed to NSCs induction medium (DMEM/F12 with Glutamax supplemented with 2% of FBS, 0.2% of Penicillin/Streptomycin, 1% of N2 supplement (100×) (Gibco, Carlsbad, CA, USA), and EGF 10 ng/mL (Gibco, Carlsbad, CA, USA)). The cells were maintained in this medium for three days, and then the medium was changed to NSCs proliferation medium (DMEM/F12 with Glutamax supplemented with 2% of FBS, 0.2% of Penicillin/Streptomycin, 2% of B27 supplement (50×) (Gibco, Carlsbad, CA, USA), EGF 20ng/mL (Gibco, Carlsbad, CA, USA), bFGF 20ng (BioLegend, San Diego, CA, USA)). The cells were cultured in this medium at 37 °C with 5% CO_2_, 21% O_2_, and 95% humidity for six days and the medium was changed every second day. The cells were then detached and dissociated with a 0.25% Trypsin/EDTA solution. Trypsin was inactivated using 2× the volume of NSCs proliferation medium, centrifuged (200× *g*, for 10 min at RT), resuspended in PBS, counted, and seeded for further immunocytochemistry analysis (a day after seeding) at a density 5 × 10^3^ cells/well in a 24-well plate. A total of 5 × 10^5^ cells were cryopreserved in 1mL of TRI Reagent (Sigma-Aldrich, St. Louis, MO, USA) for further RNA isolation. The remaining cells were used directly for immunophenotyping using flow cytometry.

### 2.4. Microscopic Characterization of Differentiated Cells (hWJ-NSCs—Human Wharton’s Jelly Derived Neural Stem Cells)

The morphology and phenotype of the cells during neural induction were observed daily under an inverted microscope (Leica, Wetzlar, Germany) with a CCD camera and analyzed using LAS V4.8 software (Leica, Wetzlar, Germany).

### 2.5. Immunocytochemistry Analysis

The immunocytochemistry analysis was performed for both undifferentiated and differentiated cells (hWJ-MSCs vs. hWJ-NSCs). Cells adhered to the 24-well plate were fixed with 4% PFA for 15 min at RT, rinsed three times with PBS, and a solution for blocking and permeabilization was added (PBS with 10% of normal goat serum (Sigma-Aldrich, St. Louis, MO, USA) and 0.2% of Triton X-100 (Sigma-Aldrich, St. Louis, MO, USA)). The cells were incubated in this solution for 1 h at RT. After washing three times with PBS, the cells were incubated overnight with primary antibodies diluted in PBS (Table 3) at 4 °C. The cells were rinsed three times with PBS to remove unbound primary antibodies and incubated with the appropriate fluorochrome-conjugated secondary antibodies (Table 3) diluted in PBS, for 1 h at RT. The cells were then washed three times with PBS to remove unbound secondary antibodies and the cell nuclei were counterstained using Hoechst 33258 (Sigma-Aldrich, St. Louis, MO, USA) for 30 min at RT.

Fluorescence microscopy was performed using an Olympus IX71 microscope and cellSens Standard V1.5 software (Olympus, Tokyo, Japan) was used for image acquisition. The same exposure time was applied for each analyzed marker.

### 2.6. Immunophenotypic Characterization of Differentiated Cells (hWJ-NSCs)

Freshly differentiated cells from 10 independent experiments were collected as described above and resuspended in PBS, counted, and stained in polystyrene tubes using fluorescently-labeled antibodies from Human Neural Lineage Kit (Cat. No 561526, BD Biosciences, San Diego, CA, USA).

For compensation, the calibrite beads (Calibrite 3 Beads, BD Biosciences, San Diego, CA, USA) were stained with each antibody conjugated to a different fluorochrome. The phenotype was characterized using a flow cytometer (FACS Aria III, BD Biosciences, San Diego, CA, USA) and Flow Jo LLC V10 software (BD Biosciences, San Diego, CA, USA). The cell populations and their phenotype were defined according to the Human Neural Lineage Kit Protocol (Cat. No 561526, BD Biosciences, San Diego, CA, USA). Four different staining variants were performed (Table 4).

### 2.7. Quantitative Reverse Transcription-Polymerase Chain Reaction (qRT-PCR)

Total RNA was extracted from cells cryopreserved in TRI Reagent (Sigma-Aldrich, St. Louis, MO, USA) using the Chomczynski method [73]. The quantity and purity of isolated RNA were measured using a NanoDrop 2000 spectrophotometer (Thermo Scientific, Waltham, MA, USA). The integrity of isolated RNA was analyzed in a 1.5% agarose gel (DNA-Gdańsk, Gdańsk, Poland) with ethidium bromide (Merck, Darmstadt, Germany).

Then, 1 µg of RNA was used in each sample duplicate (undifferentiated hWJ-MSCs: *n* = 10; differentiated hWJ-NSCs: *n* = 10) to synthesize cDNA using a commercially available kit (Transcriptor Universal cDNA Master, Roche, Basel, Switzerland). Reverse transcription was performed in the Mastercycler Nexus Gradient (Eppendorf, Hamburg, Germany). The reaction profile was as follows: incubation at 25 °C for 5 min, reverse transcription at 55 °C for 10 min, and the reaction was stopped at 85 °C for 5 min. The obtained cDNA was cryopreserved at −80 °C.

The quantitative PCR analysis was performed in duplicates per sample from 10 different umbilical cords (undifferentiated hWJ-MSCs: *n* = 10; differentiated hWJ-NSCs: *n* = 10) using LightCycler 480 SYBR Green I Master (Roche, Basel, Switzerland). The reaction was carried out in a real-time PCR system (Light Cycler 480 II, Roche, Basel, Switzerland) with Light Cycler 480 SW 1.5.1. software (Roche, Basel, Switzerland). Relative gene expression was performed using delta delta Ct (∆∆Ct) and normalized to the reference gene, *β-actin*. Undifferentiated hWJ-MSCs were used as a control. The following PCR cycling parameters were used: initial denaturation at 94 °C for 5 min; 40 cycles of 1 min at 94 °C, 1 min at 60 °C, and 1 min at 72 °C; and a final extension at 72 °C for 10 min. The primers used are listed in Table 5.

Statistical analysis was performed using GraphPad Prism V4.0 software (San Diego, CA, USA) and paired t-test. Values represent mean and standard deviation (SD) of 10 independent experiments from 10 different umbilical cords. The differences were statistically significant when *p* < 0.05. *p*-value: ** when 0.001 < *p* < 0.01, **** when *p* < 0.0001.

## 3. Results

### 3.1. Isolation, Expansion, and Immunophenotyping Characterization of hWJ-MSCs

**Figure 1:**
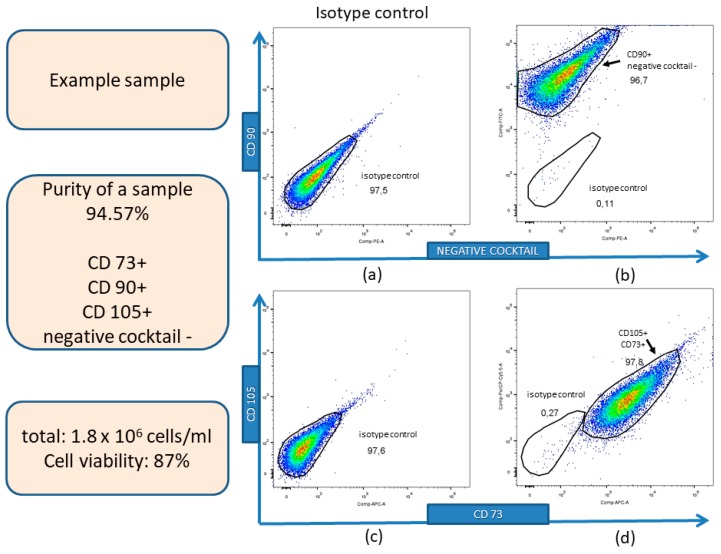
Characterization of hWJ-MSCs immunophenotype of the representative sample by flow cytometry. (**a**) Expression of CD90 and negative cocktail (CD34, CD11b, CD19, CD45, HLA-DR): an isotype control; (**b**) Expression of CD90 and negative cocktail. CD90+/negative cocktail—cells comprised 96.7% of the total cells (arrow); (**c**) Expression of CD105 and CD73: an isotype control; (**d**) Expression of CD105 and CD73. CD105+/CD73+ cells comprised 97.8% of the total cells (arrow). CD90, CD105, and CD73—markers of MSCs. The purity of presented sample defined as cell expression of CD90+, CD73+, and CD105+ and lack of expression of CD34-, CD11b-, CD19-, CD45-, HLA-DR- (negative cocktail) is 94.57%.

Isolated cells adhered to the plastic surface and displayed spindle-shaped morphology typical for MSCs. The immunophenotyping analysis of the representative sample is presented in Figure 1. Mean cell viability was 87 ± 4.56% (mean ± SD) from 10 different umbilical cords (*n* = 10) as assessed using trypan blue staining and an automatic cells counter. Cells that expressed MSCs specific markers: CD90, CD105, and CD73 but not antigens specific for endothelial and hematopoietic lineage: CD34, CD11b, CD19, CD45, and HLA-DR (negative cocktail) were defined as MSCs. The mean percentage of cells expressing the above-mentioned antigens from 10 different umbilical cords (*n* = 10) was 91 ± 3.55% (mean ± SD) and was defined as the mean purity of obtained cells.

### 3.2. Neural Induction of hWJ-MSCs

**Figure 2:**
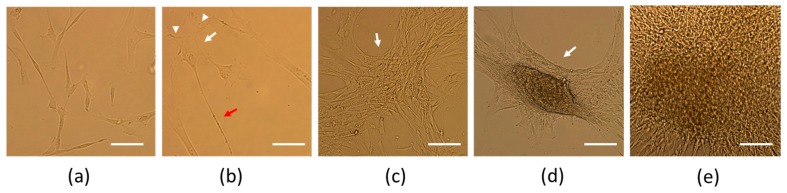
Microscopic analysis of differentiated cells (hWJ-NSCs). (**a**) Day 2 of culture, cells with spindle-shaped morphology; (**b**) day 7 of culture, cell with neural-like morphology with cell body (white arrow), processes resembling dendrites (arrowheads), and a process resembling an axon (red arrow); (**c**) day 8 of culture, cells starting to form aggregates (white arrow); (**d**,**e**) day 10 of culture, semi-adherent neurosphere-like structure (white arrow) ((**a**,**b**) scale bar = 50 µm, (**c**–**e**) scale bar = 100 µm).

Initially, differentiated cells remained adhered to the surface and maintained their spindle-shaped morphology (Figure 2a, day 2 of culture). On the fifth day of neural induction, cells with two and three poles were observed. On the seventh day, the cell morphology started to resemble neural-like cells, with a clearly visible cell body and small processes resembling dendrites and one large process resembling an axon (Figure 2b, day 7 of culture). The next day, cells started to form aggregates attached to the surface (Figure 2c, day 8 of culture). On the tenth day of differentiation these aggregates started to resemble neurospheres and started to detach from the surface (semi-adherent neurosphere-like structures) (Figure 2d,e, day 10 of culture) and the characterization of obtained cells was then performed.

### 3.3. Characterization of hWJ-NSCs

#### 3.3.1. Flow Cytometry

**Figure 3:**
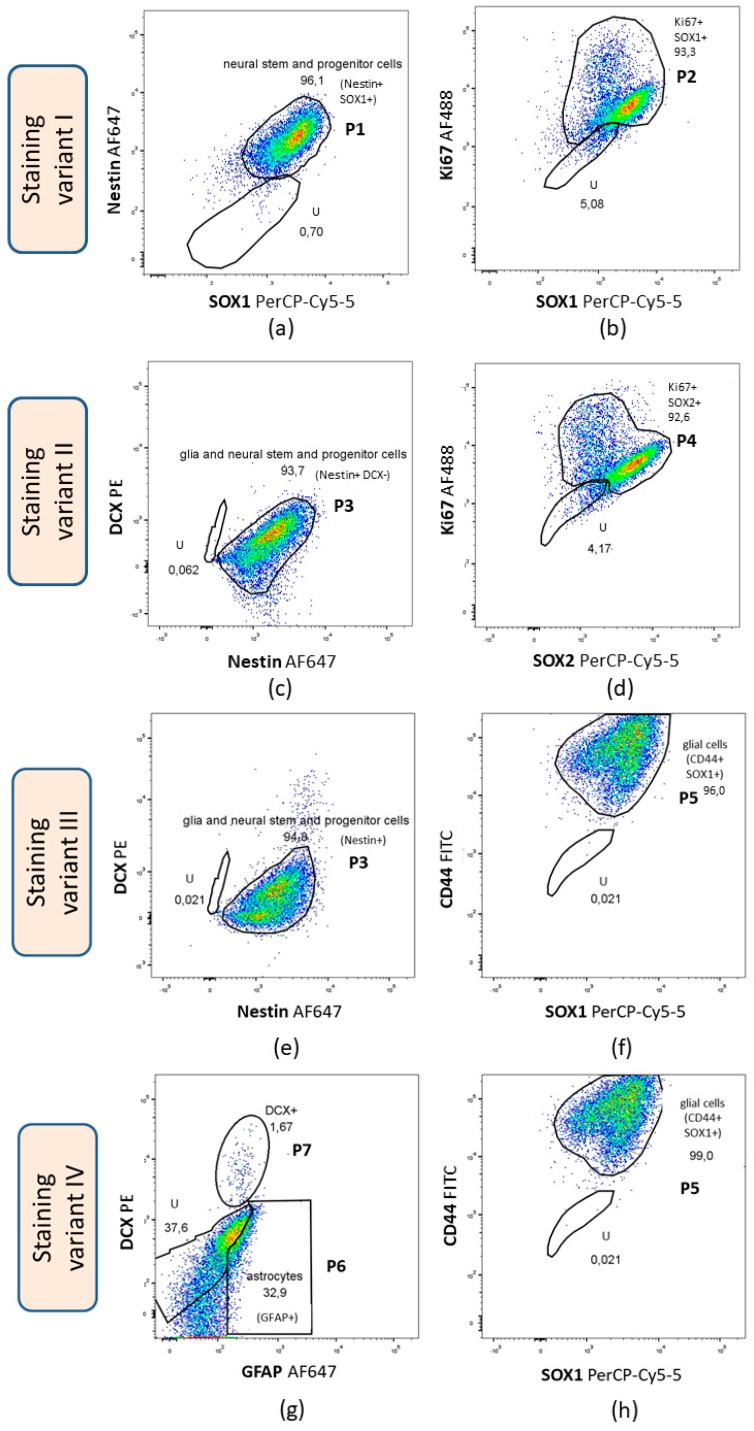
Immunophenotype characterization of the representative hWJ-NSCs sample. (**a**) Expression of Nestin and SOX1. P1 gated on the plot represents the cell population of neural stem and progenitor cells (Nestin+/SOX1+) comprising 96.1% of the total cells; (**b**) expression of Ki67 and SOX1. P2 gated on the plot represents the Ki67+/SOX1+ cell population comprising 93.3% of the total cells; (**c**) expression of DCX and nestin. P3 gated on the plot represents glia and neural stem and progenitor cells (Nestin+/DCX-) comprising 93.7% of the total cells; (**d**) expression of Ki67 and SOX2. P4 gated on the plot represents Ki67+/SOX1+ cells comprising 93.3% of the total cells; (**e**) expression of DCX and nestin. P3 gated on the plot represents glia and neural stem and progenitor cells (Nestin+/DCX−) comprising 94.8% of the total cells; (**f**) expression of CD44 and SOX1. P5 gated on the plot represents glial cells (CD44+/SOX1+) comprising 96% of the total cells; (**g**) expression of DCX and GFAP. P6 gated on the plot represents astrocytes (GFAP+/DCX−) comprising 32.9% of the total cells. P7 gated on the plot represents DCX+/GFAP- cell population comprising 1.67% of the total cells; (**h**) expression of CD44 and SOX1. P5 gated on the plot represents glial cells (CD44+/SOX1+) comprising 99% of the total cells; U—unstained cell population gated on all plots. SOX1—sex-determining region Y (SRY) box 1; SOX2—sex-determining region Y (SRY) box 2; GFAP—glial fibrillary acidic protein; DCX—doublecortin; Ki67—proliferation marker.

Several markers were analyzed using flow cytometry, such as Ki67—proliferation marker, DCX—marker of early neuronal differentiation, SOX1, SOX2, nestin—NSCs and NPCs markers, and GFAP and CD44—astroglial and oligodendroglial markers. Four different staining variants were conducted and seven different populations were gated (Table 6). Figure 3 demonstrates immunophenotypic characterization of the representative sample.

Flow cytometry analysis revealed a low percentage of cells expressing DCX (mean percentage from 10 independent experiments from different umbilical cords—4.64 ± 5.97% (mean ± SD)), whereas the mean percentage of cells expressing nestin and SOX1, defined as neural stem and progenitor cells was 96.85 ± 1.17%. The mean percentage of cells expressing SOX1 and Ki67 was 73.19 ± 30.51% and the mean percentage of cells expressing both SOX2 and Ki67—81.46 ± 17.18%. The mean percentage of cells expressing both SOX1 and CD44 was 96.35 ± 1.22% suggesting that cells displayed the phenotype of glial progenitors (astroglial and oligodendroglial). However, 32.82 ± 23.9% (mean ± SD) of cells expressed GFAP, a protein that is characteristic of astrocytes.

The obtained results indicated that a majority of the differentiated cells display the phenotype of neural stem and progenitor cells (nestin+/SOX1+). The high percentage of cells expressing CD44 and SOX1 suggests that most of the cells resemble NPCs.

The proliferation capacity of differentiated cells (hWJ-NSCs) was estimated using flow cytometry by the expression of the proliferation marker—Ki67. The mean percentage of cells expressing Ki67 was 96.1% ranging from 94.15% to 98.2%.

#### 3.3.2. Immunocytochemistry

Immunofluorescence staining of undifferentiated cells (hWJ-MSCs) confirmed the presence of CD90 (MSCs marker) and Ki67 (a marker of proliferating cells). Moreover, the low expression of nestin, SOX2 (NSCs and NPCs markers), and GFAP (a marker of NPCs and astrocytes) was observed in undifferentiated cells suggesting their high neural potential. Although a low number of undifferentiated hWJ-MSCs already expressed nestin, SOX2, and GFAP, there was an increase in the number of cells expressing these markers after neural induction.

**Figure 4:**
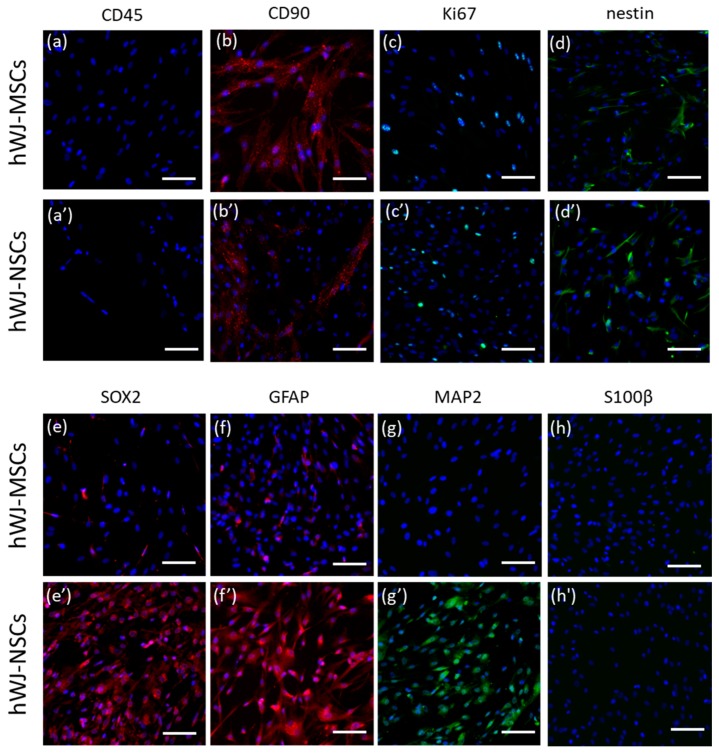
Immunofluorescence analysis of hWJ-MSCs vs. hWJ-NSCs. Comparison of expression of several markers: (**a**’) expression of CD45 (red) in hWJ-NSCs compared to (**a**) expression of CD45 (red) in hWJ-MSCs; (**b**’) expression of CD90 (red) in hWJ-NSCs compared to (**b**) expression of CD90 (red) in hWJ-MSCs; (**c**’) expression of Ki67 (green) in hWJ-NSCs compared to (**c**) expression of Ki67 (green) in hWJ-MSCs; (**d**’) expression of nestin (green) in hWJ-NSCs compared to (**d**) expression of nestin (green) in hWJ-MSCs; (**e**’) expression of SOX2 (red) in hWJ-NSCs compared to (**e**) expression of SOX2 (red) in hWJ-MSCs; (**f**’) expression of GFAP (red) in hWJ-NSCs compared to (**f**) expression of GFAP (red) in hWJ-MSCs; (**g**’) expression of MAP2 (green) in hWJ-NSCs compared to (**g**) expression of MAP2 (green) in hWJ-MSCs; (**h**’) expression of S100β (green) in hWJ-NSCs compared to (**h**) expression of S100β (green) in hWJ-MSCs; Cell nuclei counterstained with Hoechst (blue); Scale bar = 100µm. hWJ-MSCs—human Wharton’s jelly derived mesenchymal stem cells; hWJ-NSCs—human Wharton’s jelly derived neural stem cells; SOX2—sex-determining region Y (SRY) box 2; GFAP—glial fibrillary acidic protein; MAP2—microtubule-associated protein 2; S100β—S100 calcium-binding protein β; Ki67—proliferation marker.

Immunofluorescence staining of differentiated cells (hWJ-NSCs) confirmed the presence of Ki67, nestin (a marker of NSCs and NPCs), SOX2 (pluripotency marker and marker of NSCs and NPCs), MAP2 (neuronal marker), and GFAP (glial marker) in all samples. However, the low expression of S100β (a marker of mature astrocytes) in some cells was confirmed in two samples. The cells were negative for CD45 (HSCs marker), and some cells were slightly positive for CD90 (MSCs marker) (Figure 4).

**Figure 5:**
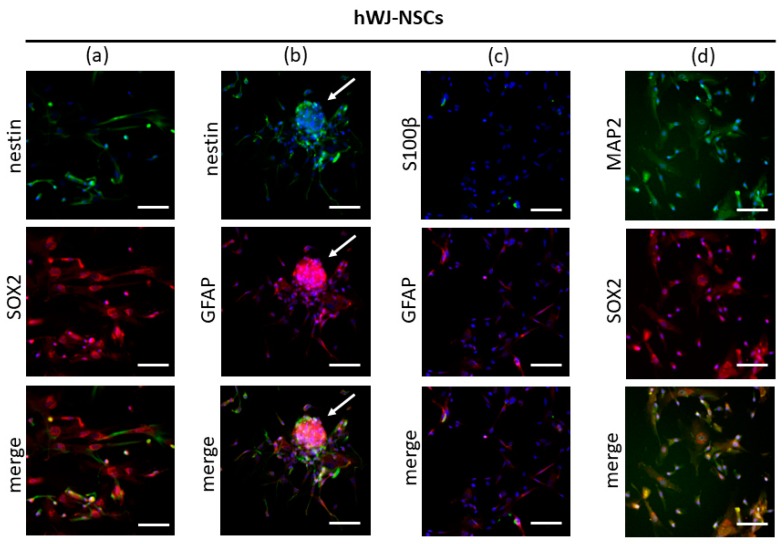
Immunofluorescence analysis of differentiated cells (hWJ-NSCs). (**a**) Co-expression of SOX2 (red) and nestin (green); (**b**) co-expression of GFAP (red) and nestin (green), neurosphere-like structure (white arrow); (**c**) co-expression of GFAP (red) and S100β (green); (**d**) co-expression of SOX2 (red) and MAP2 (green). Cell nuclei counterstained with Hoechst (blue). Scale bar = 100µm. hWJ-MSCs—human Wharton’s jelly derived mesenchymal stem cells; hWJ-NSCs—human Wharton’s jelly derived neural stem cells; SOX2—sex-determining region Y (SRY) box 2; GFAP—glial fibrillary acidic protein; MAP2—microtubule-associated protein 2; S100β—S100 calcium-binding protein β.

Double immunofluorescence analysis of differentiated cells confirmed the co-expression of nestin and SOX2 (markers of NSCs and NPCs); nestin (a marker of NSCs and NPCs) and GFAP (a marker of NPCs and astrocytes); MAP2 (a marker of mature neurons) and SOX2 (a marker of NSCs and NPCs); GFAP (a marker of NPCs and astrocytes) and S100β (a marker of astrocytes) (Figure 5a–d). Co-expression of SOX2 and nestin, as well as GFAP and nestin (Figure 5a,b), is suggestive of an NSCs/NPCs phenotype of the differentiated cells. Co-expression of mature markers (S100β or MAP2) together with markers of NSCs and NPCs (GFAP or SOX2) (Figure 5c,d) suggests that differentiated cells maintain a stem/progenitor character and do not acquire a mature phenotype.

### 3.4. Quantitative Reverse Transcription Polymerase Chain Reaction (qRT-PCR)

**Figure 6:**
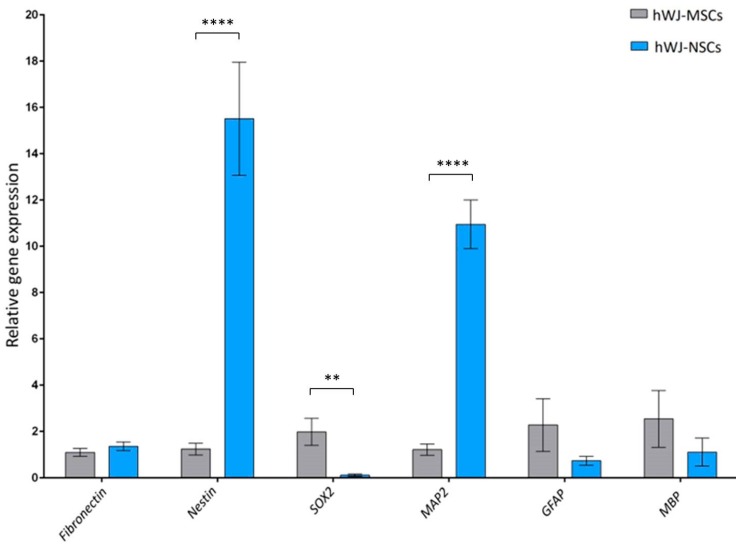
Comparison of expression of several markers on the transcript level: *fibronectin, nestin, SOX2, MAP2, GFAP, MBP* in undifferentiated cells (hWJ-MSCs) vs. differentiated cells (hWJ-NSCs) from 10 different umbilical cords. The graph represents qRT-PCR results, the relative gene expression was performed using delta delta Ct (∆∆Ct) and normalized to the reference gene *β-actin*. Bars represent mean value and SD of 10 independent experiments. Statistical analysis was performed using paired t-test. The differences were statistically significant if *p* < 0.05. *p*-value: ** when 0.001 < *p* < 0.01, **** when *p* < 0.0001. Statistically significant differences for each marker between hWJ-MSCs and hWJ-NSCs are presented using connectors. hWJ-MSCs—human Wharton’s jelly derived mesenchymal stem cells; hWJ-NSCs—human Wharton’s jelly derived neural stem cells; SOX2—sex-determining region Y (SRY) box 2; GFAP—glial fibrillary acidic protein; MAP2—microtubule-associated protein 2; MBP—myelin basic protein.

Differentiated cells (hWJ-NSCs) were analyzed on the mRNA level using reverse transcription and quantitative PCR. *β-actin* was used as a reference gene, and several genes were used as neural and glial representatives such as *nestin* and *SOX2* (markers of NSCs and NPCs), *GFAP* (glial marker), *MAP2* (neuronal marker), *MBP* (oligodendrocytes’ marker), and *fibronectin.* Fibronectin is a crucial component of the extracellular matrix (ECM) and is involved in epithelial-mesenchymal transition [74]. Moreover, MSCs can secrete fibronectin [75] promoting neurite elongation of MSCs after neural induction [76]. The obtained qRT-PCR results of hWJ-NSCs were compared to undifferentiated cells (hWJ-MSCs). The graph showing gene expression analysis is presented in Figure 6.

The data presented in Figure 6 revealed that compared to undifferentiated hWJ-MSCs, the *fibronectin* gene remained almost unchanged, whereas *nestin* and *MAP2* were greatly upregulated after differentiation. However, the *SOX2* gene was downregulated compared to undifferentiated hWJ-MSCs. The glial markers (*GFAP* and *MBP*) did not show any statistically significant difference compared to hWJ-MSCs.

In summary, the expression of the chosen NSCs/NPCs markers on transcript and protein level is similar, with some exceptions suggesting that the differentiation protocol is associated with increased expression of NSCs/NPCs markers. However, the *SOX2* gene expression was downregulated after a procedure contrary to the flow cytometry and immunocytochemistry results. The gene expression of glial markers did not increase significantly, contrary to elevated expression of those markers observed in the flow cytometry and immunocytochemistry results.

## 4. Discussion

The derivation of a stable and homogenous NSCs population remains a topic of research in many laboratories worldwide and continues to be challenging. NSCs are valuable material for studying basic science, modelling diseases, and for therapeutic purposes. As the derivation of NSCs from ESCs is related to ethical issues, using iPSCs or MSCs as a starting point seems to be an important alternative. In vitro differentiation of MSCs towards NSCs was confirmed for MSCs derived from bone marrow [40,56,66,77,78,79], adipose tissue [72,80,81,82,83], umbilical cord blood [67], Wharton’s jelly of umbilical cord [61,62,69], and amniotic fluid [84]. In this study, hWJ-MSCs with high neurogenic potential [28,61,65] and the ability to differentiate in vitro towards NSCs [61,62,64,65,68,70] were used to generate hWJ-NSCs. The presented neural induction in a monolayer culture with EGF, bFGF, N2, and B27 supplements yielded a homogenous cell population with NSC-like phenotype and the procedure was highly efficient to permit characterization of the cells using various methods. Although there are studies analyzing the expression of MSCs markers in differentiated NSCs using flow cytometry [40,85,86], the analysis of the expression of neural markers in differentiated NSCs by flow cytometry is performed very rarely [61,72] and the results are presented as separate histograms or graphs. This study provides comprehensive qualitative and quantitative flow cytometry analysis of neural and proliferation markers of NSC-like cells differentiated from MSCs. Expression of neural markers in differentiated cells is accompanied by immunocytochemistry and qRT-PCR (on protein and transcript level, respectively) analysis.

The current immunofluorescence analysis results show the expression of nestin and SOX2 in undifferentiated cells confirming their high neurogenic potential. The expression of nestin was observed previously in undifferentiated MSCs isolated from bone marrow [81] and Wharton’s jelly [58,59,61,62]. However, the expression of SOX2 was observed exclusively in undifferentiated MSCs isolated from umbilical cord blood and Wharton’s jelly, whereas the expression of SOX2 was not confirmed in MSCs from bone marrow and adipose tissue [28,58,87]. The expression of SOX2 might be altered not only by the source of the tissue but also by culture conditions and passage number [87,88]. Although comparative immunocytochemistry analysis revealed that MSCs from the umbilical cord are more likely to express SOX2, it is significantly lower than in embryonic carcinoma cells [87]. Our results show that undifferentiated MSCs from Wharton’s jelly already express neuronal and glial markers (MAP2 and GFAP). These results are in agreement with the results obtained for MSCs isolated from bone marrow and adipose tissue [81,89,90] as well as for MSCs from Wharton’s jelly [61]. Moreover, Tondreau et al. (2004) revealed that although nestin is expressed at all times in the MSCs isolated from bone marrow, the expression of GFAP and MAP2 depends on the number of passages and can be observed after the fifth passage [90].

The study aimed to develop a simple, efficient, and reproducible method of generating NSCs using a monolayer culture. A stable population of adherent NSC-like cells was obtained from ESCs [91,92] and the forebrain of an adult organism [93]. MSCs derived from bone marrow [66,69], adipose tissue [83], and Wharton’s jelly [64] were also differentiated in monolayer culture in adhesive conditions. To induce neural differentiation several different factors are used including growth factors and chemical compounds. Conti et al. (2005), similar to the current research, found that the growth and expansion of NSCs is assured by the addition of EGF and bFGF in culture [92]. In the current study, on the tenth day of culture, multipolar cells with morphology resembling neuronal cells with an axon and dendrites were observed. However, one must be careful with drawing conclusions based on cell morphology alone. Reports suggest that such changes in morphology might be caused by rapid modifications in the cytoskeleton as a result of the chemical compounds used for neural induction [94].

Cell aggregates resembling neurosphere-like structures, which spontaneously detached from the surface were observed on the last day of culture. Pollard et al. (2006) also observed the tendency of adherent adult NSCs to aggregate and form neurosphere-like structures that detached from the surface [93]. Moreover, this tendency was also confirmed during the differentiation of MSCs towards NSCs using spinal cord fluid [69]. The generation of semi-detached cell aggregates was observed at the end of the third week of hWJ-MSCs neural differentiation procedure using EGF, N2, and B27 supplements [64]. In the current study, such a tendency was observed earlier, on the tenth day of culture, suggesting that the addition of bFGF could accelerate this process. Studies have confirmed that bFGF can improve the expression of the EGF receptor (EGFR) in cell cultures [95] and modify the expression of crucial regulatory genes—SOX [93].

The differentiated cells in the current study have high proliferation capacity as was revealed in immunocytochemistry analysis through the expression of proliferation marker—Ki67. Moreover, the differentiated cells maintain the expression of MSCs marker—CD90—although it was less pronounced than in undifferentiated MSCs. Hermann et al. (2004) also observed reduced expression of MSCs markers particularly CD90 and CD166 in NSC-like cells compared to bone marrow MSCs as the starting point [86].

In the current study, differentiated cells also displayed the expression of nestin and SOX2 (NSCs and NPCs markers), GFAP (NPCs and astrocyte marker), MAP2 (a marker of mature neurons), and low expression of S100β (a marker of mature astrocytes). The presence of the above mentioned NSCs/NPCs markers suggests a phenotype of differentiated cells. Immunocytochemistry analysis revealed that the expression of nestin was comparable in both undifferentiated cells and differentiated cells. This result remains in agreement with the result obtained by Messerli et al. (2013), which confirmed a similar expression of nestin in cells forming neurosphere aggregates and in undifferentiated cells [61]. However, Leite et al. (2014) found a significantly higher expression of nestin in NSC-like cells differentiated using the indirect protocol [62]. The expression of nestin is also common among NSCs differentiated from MSCs isolated from bone marrow [40,56] and adipose tissue [80,81], regardless of the direct or indirect method used for differentiation [66,72].

In the current study, the expression of GFAP (a marker of NPCs and astrocytes) and MAP2 (a marker of mature neurons) was higher in the differentiated cells, which is contrary to the result of Messerli et al. (2013) where a difference in expression was not observed [61]. The expression of these markers was confirmed in cells forming neurosphere-like structures that were differentiated from MSCs derived from bone marrow and adipose tissue [40,56,81]. However, in the context of GFAP expression in cells differentiated in adherent culture from bone marrow MSCs, the results are inconsistent in the currently available literature. Ge et al. (2015) confirmed the presence of this protein after induction with cerebrospinal fluid [69]. However, Liu et al. (2011) did not observe the expression of GFAP after a 2D differentiation culture of rat bone marrow MSCs [66]. Moreover, many studies have found the expression of MAP2, but only after subsequent differentiation of derived NSC-like cells [56,72]. The discrepancies in the mentioned results might be a result of variable biological features of the cells according to their source, the donor, and the culture conditions [96,97]. However, a recent comparative analysis highlighted that the source of MSCs has a greater influence than the culture conditions applied for neural induction. The expression level of neural markers after common neural induction protocol was variable according to the source of MSCs [98].

In the presented data, the expression of S100β protein was almost undetectable, only two samples were slightly positive. Since S100β is a marker of mature astrocytes, expression of this protein is related to the loss of ability to generate neurospheres by the cells expressing GFAP [99]. There are only a few reports that demonstrate the expression of this protein during neural differentiation of MSCs and all of them apply to cells subsequently differentiated from MSCs-derived NSCs [72,100].

Immunocytochemistry analysis of the presented research confirmed the co-expression of nestin and SOX2, which are commonly known NSCs markers [22,23], nestin and GFAP, MAP2 and SOX2, as well as low co-expression of GFAP and S100β. GFAP is considered as a marker of mature astrocytes [101] but was found to be expressed also in NPCs [22,102]. Simultaneous expression of proteins: nestin and GFAP might suggest that differentiated cells do not acquire a phenotype of mature astrocytes and the presence of GFAP is related to their progenitor nature. This is consistent with a lack or low co-expression of GFAP and S100β that was observed. It is worth mentioning that the expression of S100β appears in cells expressing GFAP when they lose their stemness and undergo terminal differentiation [99].

The co-expression of MAP2 and SOX2 in this study suggests that the obtained cells do not undergo terminal differentiation. MAP2 is a marker of mature neurons [103], but its co-expression with transcriptional factor SOX2 suggests that the stemness of the cells is maintained. SOX2 is an important factor responsible for the self-renewal of cells and maintaining the undifferentiated character of NSCs and NPCs [27]. Although these results suggest stem/progenitor character of differentiated cells, more terminal markers would certainly be useful to exclude their terminally differentiated character.

In the presented study, the phenotype of cells was also analyzed using flow cytometry. There are not many reports involving this method for neural marker analysis of NSCs differentiated from MSCs [61,72]. The main reason for this situation might be the low efficiency of the protocol applied for differentiation leading to insufficient cell number required for flow cytometry. The presented research revealed that most of the differentiated cells express nestin, SOX1, and CD44. Nestin and SOX1 are commonly known markers of NSCs/NPCs. The mean percentage of cells expressing nestin and SOX1 was 96.85% and it corresponds with the study of Park et al. (2017) where it was demonstrated that 92.9% of cells express nestin [72]. Park et al. (2017) used a 2D culture for neural differentiation, but in addition to the growth factors, EGF and bFGF, they used small molecules such as SB431542 (Lefty/Actin/TGFβ pathway inhibitor) and noggin (an inhibitor of bone morphogenic protein) that precisely modify signaling pathways. On the other hand, Messerli et al. (2013) found that only 50% of NSCs differentiated from WJ-MSCs expressed nestin. This discrepancy could be a result of the different protocols used for neural induction. Messerli et al. (2013) used the indirect method of differentiation involving suspension culture to generate neurospheres that might cause a lower expression of nestin. Flow cytometry analysis also revealed a high percentage of cells expressing SOX1 and CD44 and these cells might represent glial progenitor cells. However, CD44 as a marker is quite unspecific and is considered as a marker of glial progenitors and especially astrocyte precursors [104] as well as a marker of NSCs and NPCs [105]. Since in the current study MSCs were used for differentiation, it has to be mentioned that CD44 is often used for MSCs characterization together with CD73 and CD90 [106]. Therefore, the presence of such a panel of markers can also hallmark NSCs or NPCs. This suggestion is supported by the studies confirming the expression of CD44 together with nestin, GFAP, S100β, MAP2, and β-tubulin III in NSCs isolated from the spinal cord of a human fetus [107] and in NSCs and NPCs derived from brain tissue of an adult human [108].

Flow cytometry analysis also revealed that GFAP and DCX were expressed on an average of 32.82% and 4.64% by differentiated cells respectively. DCX is a marker of immature neurons, whereas GFAP is considered as a marker of mature astrocytes [101] although its expression was also found in NPCs [22,102]. It should be underlined that the number of cells expressing GFAP was higher in our study compared to that of Messerli et al. (2013) [61]. This discrepancy can also be explained by the various differentiation approaches taken.

Flow cytometry analysis revealed the high proliferative potential of differentiated cells in this study; 96.1% cells on average expressed Ki67. The proliferative potential was greater than in the study of Park et al. (2017), where 76.8% of NSCs differentiated from MSCs of adipose tissue expressed Ki67 [72]. This could confirm a higher proliferative capacity of MSCs isolated from Wharton’s jelly compared to MSCs isolated from adipose tissue. The qRT-PCR analysis performed in this study revealed that differentiated NSCs have significantly higher mRNA expression of *nestin* and *MAP2* than undifferentiated MSCs. These results are consistent with those obtained by Messerli et al. (2013) and Leite et al. (2014) for neural induced MSCs isolated from Wharton’s jelly and for neural induced MSCs from bone marrow and adipose tissue [67,109,110]. Moreover, in the study by Mohammad et al. (2016), it was underlined that neural differentiation of MSCs from bone marrow in 2D culture with EGF and bFGF particularly increases *MAP2* expression, and *nestin* but to lower extent [110]. In the current study, a similar expression pattern of nestin and MAP2 was observed on transcript and protein level. However, there are discrepancies between comparative analysis of nestin between hWJ-MSCs and hWJ-NSCs on mRNA and protein level. Here, we must take into consideration that the correlation between transcript and protein abundance is relatively low in mammals and can be influenced by various biological and technical factors [111]. Significantly higher expression of nestin on transcript level suggests post-transcriptional regulation including mRNA storage in so-called processing bodies and translation blocking by mRNA-binding proteins as an essential process in protein expression [112]. Moreover, downregulated transcription of *SOX2* and *GFAP* could not be confirmed on the protein level as we observed high expression of these markers. There could be some delay between transcription and translation and therefore transcript changes will influence protein levels with a certain temporal delay. Moreover, this delay can be protein-specific due to variable transcript lengths, codon composition, or changes in translation [113]. In addition, cells adapting to a new state could first reduce the transcript levels to accelerate the transition process [114,115]. Further analysis of protein expression in subsequent passages could provide more information about the correlation between the gene and protein expression of the markers. Moreover, it is important to mention, that MSCs expanded in culture with bFGF were found to have downregulated *SOX2* mRNA suggesting bFGF as a potential factor affecting the expression of transcriptional factors [116]. Therefore, defining a cell state and phenotype solely on transcript expression can be unreliable and protein analysis has to be included.

## 5. Conclusions

Phenotypic characterization of MSCs isolated from human Wharton’s jelly confirmed the high neurogenic potential of MSCs isolated from this source. Neural induction of those cells in 2D culture yielded a stable and homogenous population of cells that could be defined as NSCs/NPCs. Furthermore, the efficiency of the proposed protocol made it possible to immunophenotype hWJ-NSCs cells using broad and comprehensive flow cytometry analysis of neural lineage markers.

In contrast to monolayer culture, the generation of a homogenous population of NSCs or NPCs in 3D culture with floating neurospheres is challenging due to the heterogeneity of neurosphere structures. However, 3D cultures maintained on alginian scaffolds [117] or in hydrogels [79] can bring breakthroughs in the field of neural differentiation in the future.

NSCs that can be differentiated from MSCs are a promising tool in the field of regenerative medicine. However, there is a need for a reliable and comprehensive comparison of their properties with endogenous NSCs that can be found in the CNS. Both direct and indirect approaches for neural differentiation can result in the generation of NSCs and NPCs. It is difficult to set a clear border between NSCs and NPCs due to the fact that both of these cell populations can be defined by a similar panel of markers [22]. Although in the present study, a feasible and efficient protocol was developed for the generation of NSCs in a 2D culture from MSCs, broader studies should be performed to check their ability of terminal differentiation towards mature neurons, oligodendrocytes, and astrocytes before translating this protocol into the clinic. Moreover, before the therapeutic application of these cells, the functional electrophysiological properties of neurons derived from NSCs have to be explored as well as the safety and effects of transplantation of these cells in animal models. After transplantation, these cells should maintain a tripotential character that would reflect their therapeutic potential.

## Figures and Tables

**Table 1:** Markers commonly used for identification of neural stem cells (NSCs), neural progenitor cells (NPCs), neurons, astrocytes, and oligodendrocytes.

Cell Type	Specific Marker	Description
NSCs	nestin	class VI cytoskeletal intermediate filament protein, essential for stem cell survival, self-renewal and proliferation, a pivotal regulator of cell differentiation and migration, can also be expressed in glial cells [24]
	PAX6	nuclear transcription factor, controls NSCs proliferation and plays a crucial role in neuronal fate determination [25]
	SOX1	transcription factor from Sox family, the earliest marker of neural fate decision [26]
	SOX2	transcription factor from Sox family, pluripotency marker, regulates neural differentiation [27], can be expressed on MSCs isolated from human Wharton’s jelly [28]
NPCs	GFAP	intermediate filament protein that serves as a holder of astrocyte mechanical strength, involved in astrocyte communication, expressed also in NPCs [22]
	PSA-NCAM	cell adhesion molecule involved in regulating several steps of adult neurogenesis [29]
	SOX1	see above
	SOX2	see above
	nestin	see above
immature neurons	DCX	protein that facilitates microtubule polymerization, a marker of migrating neuroblasts and immature neurons [30]
	βIII tubulin	microtubule protein, expressed in immature neurons and differentiated neurons [31]
neurons	MAP2	protein involved in the growth and assembly of microtubules, required for neurite formation in neurons [32]
astrocytes	GFAP	see above
	S100β	calcium-binding protein β, involved in the cell cycle and differentiation [22]
oligodendrocytes	MBP	protein involved in the myelination of neurons in the CNS [33]
	Olig1, Olig2	oligodendrocyte transcription factors [22]
	GalC	galactosylceramide expressed in differentiating oligodendrocyte precursor cells and mature oligodendrocytes [34]

SOX1—sex-determining region Y (SRY) box 1; SOX2—sex-determining region Y (SRY) box 2; PAX6—paired box 6; GFAP—glial fibrillary acidic protein; PSA-NCAM—polysialylated neural cell adhesion molecule; DCX—doublecortin; MAP2—microtubule-associated protein 2; S100β—S100 calcium-binding protein β; MBP—myelin basic protein; Olig1—oligodendrocyte transcription factor 1; Olig2—oligodendrocyte transcription factor 2; GalC—galactosylceramide; NSCs—neural stem cells; NPCs—neural progenitor cells; MSCs—mesenchymal stem cells; CNS—central nervous system.

**Table 2:** Summary of immunophenotyping analysis of mesenchymal stem cells (MSCs) isolated from human Wharton’s jelly of the umbilical cord (hWJ-MSCs).

Positive Markers	Negative Markers
CD90 CD105 CD73	CD34 CD11b CD19 CD45 HLA-DR

**Table 3:** The antibodies used in immunocytochemistry.

Specific Marker	Antibody	Concentration	Company (Cat. No)
HSCs marker	mouse anti-human CD45	1:500	BD Pharmingen (San Jose, CA, USA) (Cat. No 555480)
MSCs marker	mouse anti-human CD90	1:500	BD Pharmingen (San Jose, CA, USA) (Cat. No 555593)
proliferation marker	mouse anti-human Ki67	1:500	Merck (Darmstadt, Germany) (Cat. No MAB4190)
NSCs/NPCs marker	mouse anti-human nestin	1:250	Merck (Darmstadt, Germany) (Cat. No MAB5326)
neuronal marker	mouse anti-human MAP2	1:250	Invitrogen (Carlsbad, CA, USA) (Cat. No MA1-25044)
astrocyte marker	mouse anti-human S100β	1:250	Invitrogen (Carlsbad, CA, USA) (Cat. No MA1-25005)
NSCs/NPCs marker	rabbit anti-human SOX2	1:250	Invitrogen (Carlsbad, CA, USA) (Cat. No PA1-16968)
NPCs, astrocyte marker	rabbit anti-human GFAP	1:250	Invitrogen (Carlsbad, CA, USA) (Cat. No PA1-10019)
	AF488 goat anti-mouse IgG1	1:500	Invitrogen (Carlsbad, CA, USA) (Cat. No 845809)
	AF555 goat anti-mouse IgG1	1:500	Invitrogen (Carlsbad, CA, USA) (Cat. No 982413)
	AF568 goat anti-rabbit IgG (H+L)	1:500	Invitrogen (Carlsbad, CA, USA) (Cat. No 948491)

HSCs—hematopoietic stem cells; MSCs—mesenchymal stem cells; NSCs—neural stem cells; NPCs—neural progenitor cells; SOX2—sex-determining region Y (SRY) box 2; GFAP—glial fibrillary acidic protein; MAP2—microtubule associated protein 2; S100β—S100 calcium-binding protein β; CD—cluster of differentiation; AF—Alexa Fluor; Cat. No—catalog number.

**Table 4:** Staining variants in flow cytometry analysis of differentiated cells.

Staining Variant	Antigens Stained
**I**	Nestin, SOX1, Ki67
**II**	Nestin, DCX, Ki67, SOX2
**III**	DCX, nestin, SOX1, CD44
**IV**	DCX, GFAP, SOX1, CD44

**Table 5:** The primers used in qPCR.

Gene Name	Primer	Sequence
*β-actin*	Forward	CAGAAGGATTCCTATGTGGGC
	Reverse	GAGGGCATACCCCTCGTAGAT
*fibronectin*	Forward Reverse	GAGATCAGTGGGATAAGCAGCA CCTCTTCATGACGCTTGTGGA
*SOX2*	Forward Reverse	CAGGAGAACCCCAAGATGC GCAGCCGCTTAGCCTCG
*nestin*	Forward Reverse	CAGCTGGCGCACCTCAAGATG AGGGAAGTTGGGCTCAGGACTGG
*MAP2*	Forward Reverse	GGGCCTTTTCTTTGAAATCTAGTTT CAAATGTGGCTCTCTGAAGAACA
*GFAP*	Forward Reverse	CTGTTGCCAGAGATGGAGGTT TCATCGCTCAGGAGGTCCTT
*MBP*	Forward Reverse	CTGGGCAGCTGTTAGAGTCC TGGAGCAAAGGTTTGGTGTC

SOX2—sex-determining region Y (SRY) box 2; GFAP—glial fibrillary acidic protein; MAP2—microtubule-associated protein 2; MBP—myelin basic protein.

**Table 6:** Summary of immunphenotyping analysis of hWJ-NSCs.

Population Number	Population Name	Phenotype	Staining Variant
**P1**	neural stem and progenitor cells	Nestin+ SOX1+	I
**P2**	Ki67+ SOX1+	Ki67+ SOX1+	
**P3**	glia and neural stem and progenitor cells	Nestin+ DCX-	II, III
**P4**	Ki67+ SOX2+	Ki67+ SOX2+	II
**P5**	glial cells	CD44+ SOX1+	III, IV
**P6**	astrocytes	GFAP+ DCX-	IV
**P7**	DCX+	DCX+	
